# Bleeding us dry: The financial impact of full blood examinations in the immediate postoperative period

**DOI:** 10.1002/bco2.368

**Published:** 2024-08-13

**Authors:** Bodie Chislett, Sachin Perera, Marlon Perera, Damien Bolton, Joseph Ischia, Nathan Lawrentschuk

**Affiliations:** ^1^ Department of Urology Austin Health Melbourne Victoria Australia; ^2^ Department of Surgery Royal Melbourne Hospital, University of Melbourne Melbourne Victoria Australia; ^3^ Young Urology Researchers Organisation (YURO) Melbourne Victoria Australia; ^4^ Division of Cancer Surgery Peter MacCallum Cancer Centre Melbourne Victoria Australia; ^5^ EJ Whitten Prostate Cancer Research Centre at Epworth Healthcare Melbourne Victoria Australia

**Keywords:** cost analysis, full blood examination, haemoglobin, pathology, perioperative blood tests

## Abstract

**Introduction and objectives:**

Full blood examinations, often referred to as FBE, are commonly ordered postoperatively, despite limited utility in many of its markers in the acute phase. It is estimated that in the 2022–2023 financial year, the Australian healthcare system billed over $13 million for full blood examinations (FBEs) to Medicare. This study aims to assess the cost of using FBE following surgery. We explore potential cost savings by using a haemoglobin examination (HE) in replace of FBE, with both tests run on identical machines, producing the same result, but at a fraction of the cost.

**Methods:**

A retrospective analysis was conducted at a single institution, including all patients who underwent minimally invasive laparoscopic pelvic surgeries between 1/7/2017 and 30/6/2019. Patient records were examined to identify patient demographics, postoperative pathology tests and interventions. Patients who received FBE in the first 24 h following surgery were identified and included in the study. Using national surgery and admission statistics, a potential cost‐saving analysis will be performed.

**Results:**

Among 519 men who underwent robotic‐assisted pelvic surgery, 325 patients had routine postoperative investigations, with 323 receiving FBE and 2 receiving HE. Abnormal results were found in the majority of patients that underwent FBE. Eight patients received packed red blood cell transfusion, none of these meeting the hospital‐specific criteria for transfusion protocol. Twelve patients received antibiotics, none were in response to abnormal FBE, with all patients experiencing a fever, given prophylactically or in the days following the surgery. FBE and HE are both listed on the Medicare Benefits Scheme at $16.95 and $7.85, respectively, the difference being $9.10. Extrapolating the existing data, within the first 24 h following surgery, the estimated savings were $8818, with savings increasing accumulatively with longer observation intervals following surgery. When similar savings are applied to national figures, the potential savings to the Australian Public Healthcare system likely exceeds millions.

**Discussion:**

Our study revealed that over half of the patients who underwent a RARP received FBE within the first 24 h postoperatively, the vast majority of which exhibited abnormal results that were not acted upon. These findings substantiate the limited utility of FBE in the postoperative setting. Cell markers observed in FBE are predominantly subjective, but consensus exists regarding the importance of evaluating haemoglobin levels postoperatively. Considering that one in four hospital admissions involves surgical procedures, and a $9.10 price differential between FBE and HE, the potential annual economic impact of utilising routine FBEs for assessing haemoglobin levels immediately after surgery is likely to reach millions. Although having obvious flaws, these results underscore the potential accumulative cost arising from everyday clinical judgement and the importance of thoughtful consideration when ordering pathology.

**Conclusion:**

The routine ordering of FBE postoperatively, without properly considering its indication, incurs significant costs. This study highlights the potential cost savings by HE instead, emphasising the need for revaluation and appropriate utilisation of pathology tests in the immediate postoperative period given the physiological acute phase response expected postoperatively.

## 1 INTRODUCTION

Pathology tests play a critical role in modern healthcare, providing valuable insights into patients' health and guiding clinical decision making. However, the indiscriminate use of such tests can lead to unnecessary healthcare expenditures and potential harm to patients. The ‘Choosing Wisely’ campaign, originally established by the American Board of Internal Medicine, seeks to address this issue by promoting conversations among clinicians about the rational use of medical resources.^1^ This paper focuses on the application of ‘Choosing Wisely’ principles in the context of full blood examinations (FBEs) following surgery, examining their utility and proposing a cost‐effective alternative.

FBEs, also known as complete blood counts or full blood counts, are tests that assess various components of whole blood, including haemoglobin, red blood cells, various white blood cells and platelet counts. However, the clinical value of most FBE parameters in the immediate postoperative period is limited. The expected physiological responses to surgery leads to significant fluctuations, in particular, inflammatory and infective markers like white blood cells are expected to rise. This means their use for assessing underlying pathological processes is obscured by surgery, difficult to interpret and offers limited utility. Consequently, the only valuable postoperative marker provided by FBEs is the haemoglobin (Hb) count. Haemoglobin examinations (HEs) offer a reliable and cost‐effective alternative for testing Hb levels, with both FBE and HE being performed on the same machine, under the same conditions, with HE being half the cost according to the Australian ‘Medical Benefits Scheme’.^2^

This study aims to assess the frequency of FBE use postoperatively while examining its utility through the frequency of interventions that are attributed to abnormal FBE results. A rudimentary cost analysis will be performed through the adoption of HE in place of FBE.

## 2 METHODS

A retrospective study will examine patients who underwent robotic‐assisted radical prostatectomy (RARP) at an Australian institution, over 2 years from 29 June 2017 to 28 June 2019. A single clinician will review medical records and register patient demographics, postoperative FBE and HE use and all abnormal results based on the hospital's specific range.

Using a combination of procedural codes and a review of medical records, the study will evaluate the frequency of clinical interventions considered to be attributable to abnormal FBE results, including packed red blood cells, platelet transfusions and antibiotic use. Using the ‘Medical Benefits Scheme’ pricing for FBE ($16.95) and HE ($7.85), a rudimentary cost analysis will be performed to assess potential savings if FBE use were to be replaced by HE within the first 24 h following surgery.^2^ This cost analysis will be extended to a national scale, to reflect the volume of surgery performed within Australia by using statistics from the 2022–2023 Australian Department of Health report.

## 3 RESULTS

A total of 519 cases were identified, with 323 patients receiving postoperative FBE within the first 24 h. The remaining 194 cases were discharged without undergoing postoperative blood tests. A significant proportion of FBE results (81%) were abnormal, primarily due to deranged white blood cell counts. A total of 20 interventions were recorded, the majority occurring in patients who underwent postoperative FBE (18/20) while the remaining two were in patients who did not receive postoperative tests. Eight patients received blood transfusions, none meeting the hospital‐specific transfusion criteria. No patients received platelet transfusions. Antibiotics were administered to 12 patients, primarily in response to fever or prophylactically, none were documented to be in response to abnormal FBE results. A total of 7% (25) patients had abnormal platelet counts, none less than 100 × 10^9^/L or requiring transfusion. No HE examination returned abnormal results.

**FIGURE 1:**
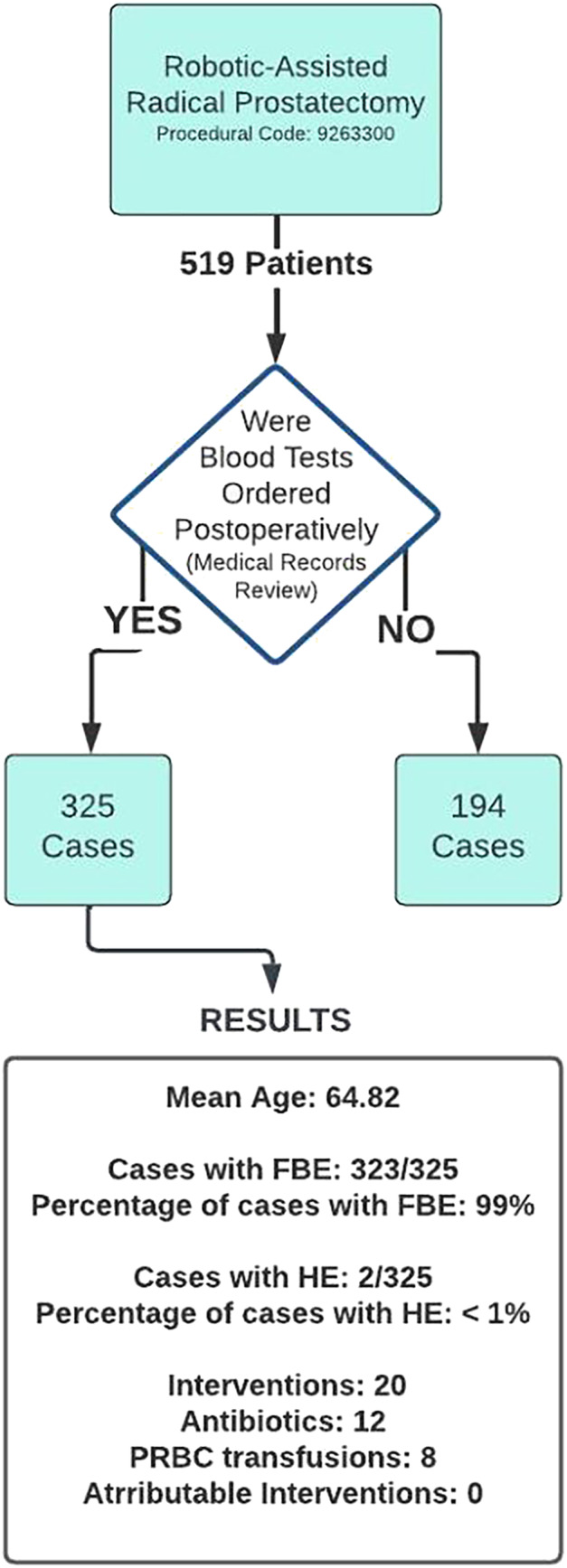
Flow diagram of methodology and results.

Cost analysis was performed using the current ‘Medical Benefits Scheme’ pricing schedule for FBE ($16.95) and HE ($7.85), both being unchanged from 2018. Applying the cost differential between FBE and HE ($9.10), a saving of $2939 could be saved on 323 patients. In the 2021–2022 financial year, 374 000 emergency hospital admissions involved surgery and 2.3 million admissions for elective surgery.^3^ Using a conservative estimate of 10% of cases undergoing postoperative FBE, the potential annual windfall nationally for modifying practice habits equates to AUD 2.4 million (Figure 1).

## 4 DISCUSSION

Our study revealed that over half of the patients who underwent a RARP received FBE within the first 24 h postoperatively, the vast majority of which exhibited abnormal results that were not acted upon. Of those patients who received postoperative blood tests, 99% of patients underwent FBE, with 81% returning abnormal results. The vast majority of those that returned abnormal results were a result of deranged white blood cell counts, a known response to surgery. We were unable to identify any patient who received postoperative antibiotics due to abnormal FBE results. These findings substantiate the limited utility of FBE in the postoperative setting for assessing infection.

The utility of cell markers observed in FBE is predominantly subjective, but consensus exists regarding the importance of evaluating haemoglobin levels postoperatively. Haemoglobin levels serve as an indication of blood loss, continued bleeding and need for transfusion. Assessing for postoperative anaemia can be cost‐effectively achieved using HE. Estimating the economic impact of FBE use in the postoperative context proves challenging due to limited national data concerning the timing of investigations used concerning patient admissions. However, considering that one in four hospital admissions involves surgical procedures, and a $9.10 price differential between FBE and HE, the potential annual economic impact of utilising routine FBEs for assessing haemoglobin levels immediately after surgery is likely to reach millions. Our findings underscore these estimations, with projected savings for 10% of cases exceeding 2 million AUD from the adoption of HE in lieu of FBE. Although having obvious flaws in this assessment, these results underscore the potential accumulative cost arising from everyday clinical judgement and thoughtful consideration of routine pathology practices.

The cause for over utilisation of pathology tests in general is likely multifactorial. One contributing factor is the development of routine behaviours in medical practice, wherein clinicians forego deliberation and cease questioning the utility of investigations. This has led to the adoption of vernacular such as ‘routine bloods’. Furthermore, it is often inexperienced practitioners, particularly junior doctors, who are granted the autonomy and responsibility for ordering pathology investigations. A study by Miyakis et al. demonstrated that junior medical staff were 20% more likely to order inappropriate investigations compared with their more senior counterparts.^4^ A clinical understanding of the costs associated with specific investigations can also play a role in pathology overutilisation, where presenting fee data alongside frequently ordered pathology tests has been shown to effectively reduce the volume of ordered tests by approximately 8%.^5^ Additionally, fear of litigation constitutes another driving force behind the overuse of resources, akin to routine practice habits and staff inexperience. The apprehension of potential litigation induces clinical uncertainty among practitioners, compelling them to order a higher number of tests in an attempt to safeguard their professional interests.

## 5 CONCLUSION

This study underscores the need for a more rational approach to the use of FBEs in the immediate postoperative period. The majority of FBE cell counts are irrelevant during this phase due to the body's acute inflammatory response to surgery. The study suggests that replacing FBEs with HEs, which focus on haemoglobin levels, is a cost‐effective alternative. The potential financial implications of inappropriate FBE use are significant, highlighting the importance of thoughtful consideration of routine pathology practices. Strong leadership, specific mandates and education are essential to address wasteful resource use and promote more responsible clinical practices.

## AUTHOR CONTRIBUTIONS

**Bodie Chislett:** conceptualisation/writing. **Sachin Perera:** writing. **Marlon Perera:** supervision. **Damien Bolton:** supervision. **Joseph Ischia:** supervision. **Nathan Lawrentschuk:** supervision/review.

## CONFLICT OF INTEREST STATEMENT

There are no conflicts of interest to be disclosed.
